# *Acinetobacter calcoaceticus-baumannii* complex prevalence, spatial-temporal distribution, and contamination sources in Canadian aquatic environments

**DOI:** 10.1128/spectrum.01509-24

**Published:** 2024-09-06

**Authors:** Thomas Benoit, Dania Sajjad, Michel Cloutier, David R. Lapen, Emilia Craiovan, Ellen M. E. Sykes, Ayush Kumar, Izhar U. H. Khan

**Affiliations:** 1 Ottawa Research and Development Centre, Agriculture and Agri-Food Canada, Ontario, Canada; 2 Department of Chemistry and Biomolecular Sciences, University of Ottawa, Ontario, Canada; 3 Department of Microbiology, University of Manitoba, Winnipeg, Manitoba, Canada; Connecticut Agricultural Experiment Station, New Haven, Connecticut, USA

**Keywords:** *Acinetobacter calcoaceticus-baumannii *complex, agriculture, surface water, environment, wastewater, beach water

## Abstract

*Acinetobacter calcoaceticus-baumannii* (ACB) complex has been identified as a group of emerging opportunistic pathogens that cause nosocomial infections. The current study investigates the prevalence, distribution, and diversity of pathogenic ACB complex in various aquatic systems with different uses. Of the total 157 agricultural, raw drinking water intake, recreational beach, and wastewater treatment plant (WWTP) effluent samples, acinetobacters were isolated, quantified, and confirmed by genus- and ACB complex-specific PCR assays. Of all agricultural surface water samples, *A. calcoaceticus* (65%) was more frequently detected than *A. pittii* (14%), *A. nosocomialis* (9%), and *A. baumannii* (3%). In WWTP effluent samples, *A. baumannii* was more prevalent in de-chlorinated (60%) samples compared to both *A. pittii* and *A. nosocomialis* (40%). Interestingly*, A. nosocomialis* (43%), *A. calcoaceticus* (29%), and *A. baumannii* (14%) were detected in raw drinking water intake samples, whereas *A. pittii* (50%) and *A. nosocomialis* (25%) were detected in beach samples. Although no sampling location-specific differences were recorded, significant (*P* < 0.05) seasonal differences were observed when agricultural surface water samples collected in spring were compared with the summer and fall. Whereas effluent chlorination significantly impacted the degree of prevalence of *Acinetobacter* in WWTP effluent samples, overall, the prevalence of ACB complex in all sampling locations and seasons indicates that these water sources, containing human-associated ACB complex, may pose potential health risks as community-acquired opportunistic infections.

**IMPORTANCE:**

*Acinetobacter calcoaceticus-baumannii* (ACB) complex is a group of organisms known to cause problematic nosocomial opportunistic infections. A member of the species complex, *A. baumannii*, is becoming a global threat to infection treatment as strains are increasingly develop resistance to antibiotics. The prevalence and distribution of potentially pathogenic *Acinetobacter calcoaceticus-baumannii* complex species remain poorly understood, and there is a need to better understand the occurrence of *A. baumannii* in non-nosocomial environments. Our research details the spatial-temporal distribution of ACB complex species in a regional watershed and highlights the presence of ACB complex in wastewater effluent that is discharged into a river. These findings deepen our understanding of this group of species in non-nosocomial environments and encourage the development of monitoring programs for these species in regional waters.

## INTRODUCTION

Aquatic environments may become contaminated with human-associated bacterial pathogens through sewer and septic leakage, seepage, sewer overflows, agricultural runoff, and treated wastewater discharge (1, 2). *Acinetobacter* species have been found in a wide range of environments, including water and soil. They were considered low-virulence pathogens of minimal significance until the late 1960s when infections started to emerge (3–5). Among several *Acinetobacter* species, the *Acinetobacter calcoaceticus-baumannii* (ACB) complex, including *A. baumannii*, *A. pittii*, and *A. nosocomialis*, was identified as opportunistic pathogens and commonly linked to pneumonia, bacteremia, wound, and catheter infections with antibiotic resistance properties (6, 7). Coinciding with the increasing use of antibiotics, extensively resistant strains of *A. baumannii* have since been identified (8). There is now an increase in cases of *Acinetobacter* infections and deaths in the United States, underscoring *A. baumannii*'s relevance as a predominant nosocomial pathogen (9).

The genus *Acinetobacter* is described as ubiquitously prevalent in the environment, with certain ACB complex species such as *A. calcoaceticus*, *A. nosocomialis*, and *A. pittii* having previously been detected in soil and water habitats. However, *A. baumannii* is often associated with human nosocomial environments and has not been well-defined in environments with low human impact (10–12). Infections caused by the ACB complex are difficult to treat due to the development of multidrug resistance mechanisms within these species (13). *A. baumannii* has been described as clinically significant due to its ability to rapidly develop antimicrobial resistance and survive for long periods of time in unfavorable environments (14, 15). Although the natural reservoir of *A. baumannii* remains undefined, *A. baumannii* and other ACB complex have been reported outside the hospital setting in various environmental sources ranging from soil, water, wastewater, manure, and agricultural crops (16, 17).

In recent years, researchers have investigated the prevalence of *Acinetobacter* species in untreated, treated wastewater, and activated sludge (18–20). Chlorination is the most commonly used method for treating wastewater effluent in North America through primary, secondary, and tertiary stages (21). However, it has been reported that the chlorination strategy can selectively promote the survival of antibiotic-resistant bacterial (ARB) species such as *Pseudomonas aeruginosa* (22). Chlorination also results in the presence of free and combined chlorine residues, which are toxic to aquatic habitats in the receiving waterbodies; as such, many wastewater treatment plants (WWTPs) are required to de-chlorinate the treated wastewater prior to discharge into the environment (23, 24). Therefore, receiving watersheds could be reservoirs for emerging waterborne pathogens.

The discovery of *A. baumannii* in a regional watershed sample posed significant questions as to the prevalence of the pathogen in natural waterways and potential sources of contamination (25). In this study, we investigated the rate of prevalence, distribution, and potential contamination sources of the ACB complex in various aquatic environments including agricultural, recreational, and agriculturally impacted raw drinking water intake sources. As *A. baumannii* is often identified in human-impacted environments, discovery of this pathogen and the ACB complex prompted further investigation into potential contamination sources of these waters. Therefore, we also investigated the prevalence and distribution of these pathogens in a WWTP. The investigation included pre-chlorinated, chlorinated, and de-chlorinated effluent to assess the effectiveness of the treatment strategy on the prevalence and load reduction of ACB complex and other *Acinetobacter* species in the treated effluent. This investigation will better improve or develop strategies to protect aquatic environments and reduce human health risks.

## MATERIALS AND METHODS

### Study site description and water sampling

**Fig 1:**
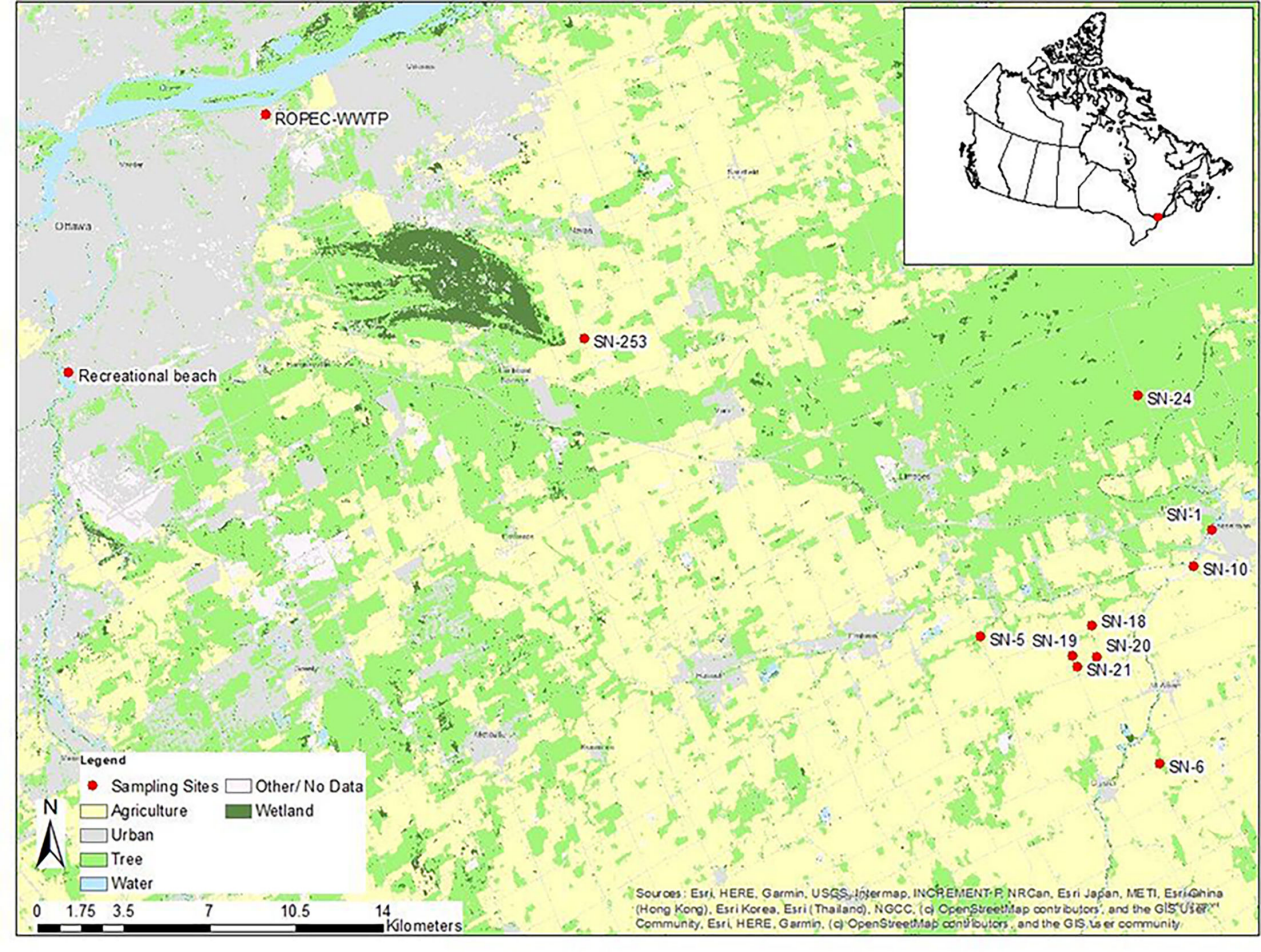
Study map showing various aquatic sampling sites including agricultural tributary (SN-5, SN-6, SN-10, and SN-253) and drainage (SN-18, SN-19, SN-20, and SN-21), forest (SN-24), drinking water intake source (SN-1), wastewater treatment plant (ROPEC-WWTP), and recreational beach.

The present study was focused on various aquatic environments, such as agricultural drainages (water channels draining tile water from fields), rivers and tributaries, streams (draining a non-agricultural forested area), a municipal beach, a raw drinking water intake, and three wastewater treatment effluent stages. All water samples were collected in sterile 1 L polypropylene bottles and placed on ice for transport to the laboratory where samples were processed for the isolation of *Acinetobacter* species within 24 hours of their collection. Sampling locations are detailed in Fig. 1.

A total of 127 surface water samples were collected, on a biweekly basis from nine sites, between May and October of 2018 and 2019, in the South Nation River (SNR) watershed, eastern Ontario, Canada. The SNR watershed covers approximately 4,000 km^2^ and about 60% of the land is under agriculture and livestock usage (26). Dairy and livestock farming are dominant land use in the area and many fields receive manure applications in the spring and fall. The watershed designation, site description, and stream order detail have previously been documented and are summarized in Table 1 (27–30).

**TABLE 1:** Description of sampling site locations

Location	Site	Description
	SN-5	River with a dairy operation upstream of the sampling site.
Tributary	SN-6	River that drains predominantly cropland area with nearby dairy operations
	SN-10	Creek with a former dairy operation directly upstream.
	SN-253	Tributary that drains predominantly cropland area with some expanding urban development upstream.
	SN-18	Manmade agricultural drain of a cropland area fed by sub-surface tile drainage.
	SN-19	Manmade agricultural drain of a cropland area fed by sub-surface tile drainage.
Drainage	SN-20	Manmade agricultural drain of a cropland area fed by sub-surface tile drainage. Dredged in November 2018 to restore flow capacity of the drain.
	SN-21	Manmade agricultural drain of a cropland area fed by sub-surface tile drainage. Dredged in November 2018 to restore flow capacity of the drain.
Stream	SN-24	Non-agricultural primarily forested region with wetlands in the vicinity as well as forest management operations. Proximal to former logging roads and recreational trails.
Raw drinking water	SN-1	Drinking water treatment plant (WTP) intake source along the SNR with residential and commercial properties adjacent to the site.
Beach	Ankle	Surface water collected on the beachfront at ankle depth. Recreational, residential, and commercial activities are directly upstream
	Knee	Surface water collected on the beachfront at knee depth. Recreational, residential, and commercial activities are directly upstream
ROPEC	Pre-chlorinated	Secondary clarification tank water representing the pre-chlorinated treatment phase.
	Chlorinated	Chlorination treatment effluent tank representing the chlorinated treatment phase.
	De-chlorinated	De-chlorination treatment effluent tank representing the de-chlorinated treatment phase.

During the 2019 water recreational season between July and August, four (two of each from ankle and knee depth zones) surface water samples were collected from a recreational beach located on the Rideau River, Ottawa, Ontario. Recreational, residential, and commercial activities are directly upstream.

In addition, a total of 30 samples, on a biweekly basis, were collected from the Robert O. Pickard Environmental Centre's wastewater treatment plant in Ottawa, Ontario, from three treatment phases to assess the chlorinated wastewater-treated effluent. This plant has an average capacity of 545 million liters per day and provides secondary municipal wastewater treatment to approximately 1,000,000 PE (population equivalent). The wastewater originates from inputs from domestic, hospital, industrial and stormwater sources. Sampling occurred on secondary clarification tank water representing the pre-chlorinated treatment phase. The second and third samplings were from chlorinated and de-chlorinated effluent tanks prior to the treated wastewater discharge to the Ottawa River.

### Sample processing and culturing conditions for isolation of *Acinetobacter* species

All water samples were processed by membrane filtration using 0.45 µm mixed cellulose ester membrane filters (PALL Corporation, New York, USA). Based on relative turbidity, a volume between 1 and 100 mL was filtered. For the enhanced recovery and broad detection of *Acinetobacter* and ACB complex species, filters were placed on CHROMagar Acinetobacter (CHROMagar, Paris, France) and Leeds Acinetobacter Medium (HiMedia Laboratories Pvt. Ltd., Mumbai, India) *Acinetobacter* selective and differential growth media, and plates were incubated at 30, 37, and 42°C, under aerobic and microaerobic conditions as previously described (31). Based on colony morphology of *Acinetobacter* species observed on CHROMagar and LAM (Fig. S1) media plates, putative *Acinetobacter* cultures were quantified for cell concentration using a single incubation condition. Putative colonies were purified on selective growth media and further confirmed by genus and species-specific PCR assays.

### DNA extraction

DNA extraction for the putative *Acinetobacter* cultured isolates was performed using previously described single colony boiling method (32). Briefly, a single colony in suspended 100 µL TE (10 mM Tris-HCl, pH 8.0, 1 mM EDTA) buffer was boiled for 10 min and briefly centrifuged. Extracted DNA was quantified using a Qubit 3.0 fluorometer (Thermo Fisher Scientific, USA) and stored at −20°C for PCR analysis.

### Genus- and species-specific PCR amplification assays

For the detection of *Acinetobacter* species to genus level, all putative culture isolates were subjected to PCR using genus-specific PCR primers as listed in Table 2 (33–37). PCR amplification was carried out in a Mastercycler EP gradient thermal cycler (Eppendorf, Germany) with a 25 µL reaction mixture containing 10–20 ng of DNA template, 1 U of Fermentas Taq DNA polymerase, 1 × buffer containing MgCl_2_ and KSO_4_, 200 µM each of the dNTPs (Fisher Scientific, Canada), and 0.4 µM each of forward and reverse primers by adjusting the volume to 25 µL with sterile distilled water. The PCR reaction was performed using an initial template denaturation step at 94°C for 3 min, followed by 35 cycles of amplification (denaturation at 94°C for 30 s, annealing at 58°C for 30 s, and elongation at 72°C for 90 s), ending with a final extension at 72°C for 10 min.

**TABLE 2:** *Acinetobacter* genus- and species-specific PCR primer sequences used for the study

Target species	Target gene	Primers	Sequences (5′−3′)	Amplicon size (bp)	Reference
*Acinetobacter* spp.	*rpo*B	Ac696F	TAY CGY AAA GAY TTG AAA GAA G	900	(33)
		Ac1598R	CGB GCR TGC ATY TTG TCR T		
		Ac1055F	GTG ATA ARA TGG CBG GTC GT		
		Ac1093R	CMA CAC CYT TGT TMC CRT GA		
*A. baumannii*	ITS	P-Ab-ITSF	CAT TAT CAC GGT A AT TAG TG	208	(37)
		P-Ab-ITSB	AGA GCA CTG TGC ACT TAA G		
*A. pitti*	ITS	P-AGS3-F	CTC AAG AGT TTA GAT TAA GCA AT	150	(36)
		P-AGS3-R	GTC CGT GCG ATT CTT CAT CG		
*A. baumannii* and *A. nosocomialis*	*gyr*B	sP4F	CAC GCC GTA AGA GTG CAT TA	294	(34)
		sP4R	AAC GGA GCT TGT CAG GGT TA		
*A. calcoaceticus*	*rpo*B	Acal-F	TCG TAT CTC AAT TAC ACC GTT CAC CT	549	(35)
		Acal-R	CGC CTT CTG CCA GTT TCA CCA TA		

Isolates positive for *Acinetobacter* species were further investigated for ACB complex differentiation using species-specific mono- and multiplex PCR assays. The PCR primers and protocol were performed as previously described (36, 37) to identify *A. baumannii*, *A. nosocomialis*, and *A. pittii* isolates targeting *gyr*B.*rec*A and 16S-23S rRNA internal transcribed spacer region genes. The PCR reaction was performed in a 25 µL reaction mixture containing 10–20 ng of DNA template, 1 U Fermentas Taq DNA polymerase, 1 × buffer, 200 µM each of the dNTPs (Fisher Scientific, Canada), 0.4 µM each of *A. baumannii* and *A. nosocomialis*, and 0.1 µM *A. pittii* forward and reverse primers (Table 2) using an initial template denaturation step of 94°C for 5 min, followed by 35 cycles of amplification (denaturation at 94°C for 1 min, annealing at 58°C for 30 s, and elongation at 72°C for 30 s) ending with a final extension at 72°C for 10 min.

For the identification of *A. calcoaceticus*, species-specific PCR amplification was carried out as previously described (35). The PCR reaction was performed in a 25 µL reaction mixture containing 10–20 ng of *A. calcoaceticus* DNA template, 1 U Fermentas Taq DNA polymerase, 1 × buffer, 200 µM each of the dNTPs (Fisher Scientific), and 0.4 µM each of forward and reverse primers (Table 2) using an initial template denaturation at 95°C for 5 min, followed by 30 cycles of amplification (denaturation at 95°C for 20 s, annealing at 68°C for 30 s, and elongation at 72°C for 30 s) ending with a final extension at 72°C for 7 min. In each PCR reaction, ACB complex reference strains and other closely related *Acinetobacter* species were used as positive and negative controls.

Based on the amplicon size, all PCR reactions were electrophoresed on a 1% or 1.5 % agarose gel matrix and stained in ethidium bromide (0.5 µg mL^−1^). The expected amplicon size of each target species was confirmed by using a 100 bp DNA size marker (Fisher Scientific). The gels were visualized on an ultraviolet (UV) transilluminator and photographed using an Alpha Imager (Fisher Scientific) gel documentation system.

### Statistical data analysis

Prevalence data were comparatively analyzed using contingency tables and Chi-squared test for statistical significance. Seasonal concentration data for agricultural water samples were analyzed by applying two-way analysis of variance (ANOVA) with Tukey's multiple comparisons test when comparing multiple means to each other. Concentration data for wastewater treatments were analyzed by one-way ANOVA with Tukey's multiple comparisons test. All data were analyzed and presented using GraphPad Prism (version 8.0) software. Statistical significance was determined as *P* < 0.05 (*).

## RESULTS

### *Acinetobacter* species prevalence and cell concentration in agricultural surface water

**Fig 2:**
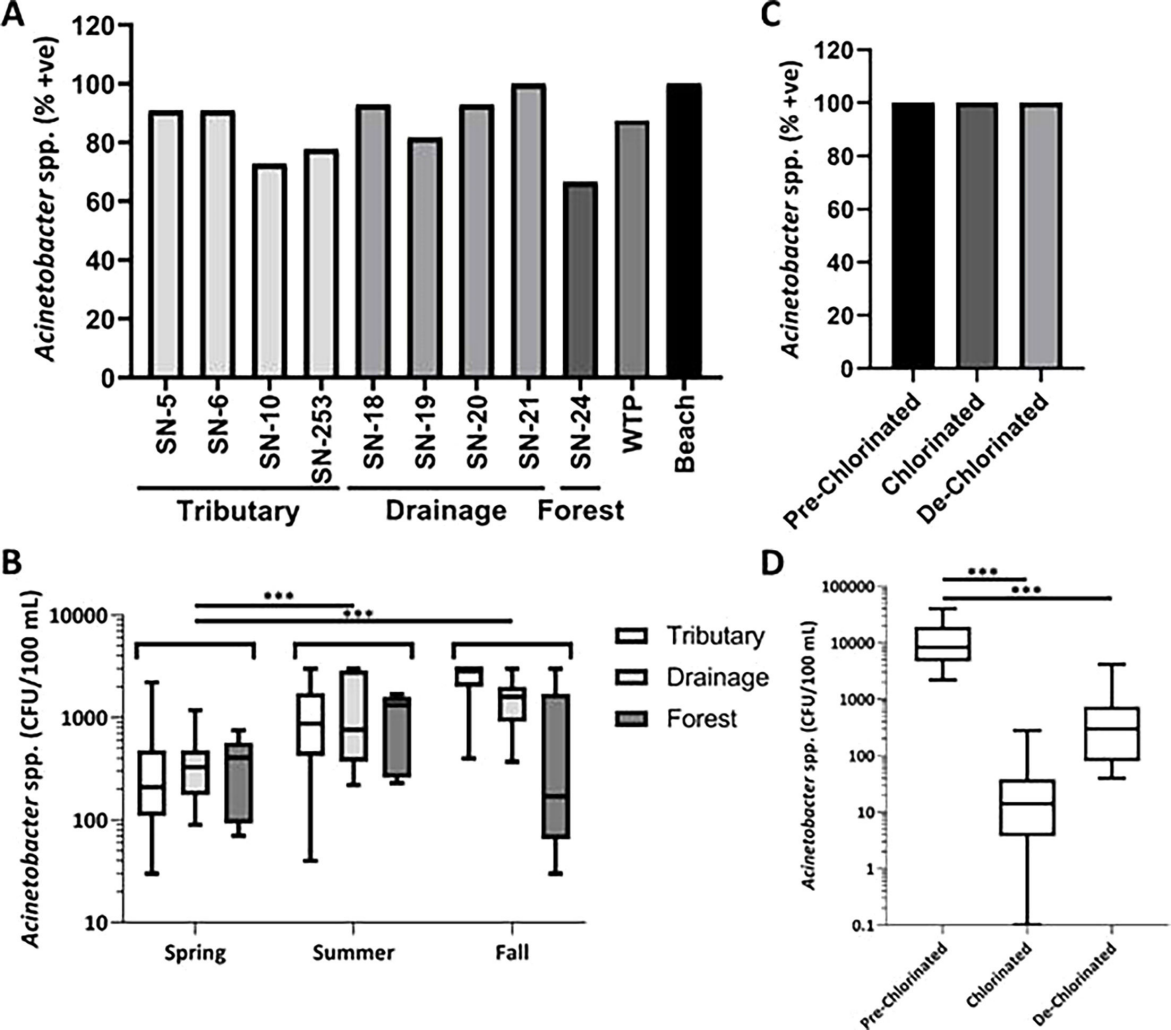
Percent *Acinetobacter* species-positive samples collected from various aquatic environments. (A) prevalence of *Acinetobacter* species in agricultural, forest, raw drinking intake, and beach surface water samples. (B) season volume-weighted average cell concentrations (CFU 100 mL^−1^) of *Acinetobacter* species from agricultural (Tributary: 5, 6, 10, and 253; Drainage: 18, 19, 20, and 21) and forest/wetland (24). (C) prevalence of *Acinetobacter* species in WWTP treatment stages. (D) volume-weighted average concentrations (CFU 100 mL^−1^) of *Acinetobacter* species from WWTP pre-chlorinated, chlorinated, and de-chlorinated samples. Statistical significance (*P* < 0.001) is shown as *** in (B) and (D).

Based on the *Acinetobacter* genus-specific PCR assay, of the total 115 agricultural surface water samples, *Acinetobacter* species were frequently (83%) detected at all sampling sites. Notably, all sampling locations were positive for *Acinetobacter* species, and site-specific examination revealed frequent detections: SN-21 (100%), SN-18 and SN-20 (93%), SN-5 (91%), SN-6 (91%), SN-19 (82%), and SN-253 (78%). Interestingly, sites SN-10 (73%) and SN-24 (forest reference site) (67%) had comparatively lower prevalence relative to other sampling locations, but no significant (*P* < 0.05) differences were observed compared to these sites (Fig. 2A). Meanwhile, the WTP intake (SN-1) site also had a high prevalence (88%) of *Acinetobacter* spp. in the samples examined. *Acinetobacter* species were also detected in all ankle- and knee-depth beach water samples (Fig. 2A). Further analysis examined the cell concentration and rate of occurrence based on seasons in agricultural water samples. As determined by the described colony morphology on selective media, the overall volume-weighted average of putative culturable *Acinetobacter* species concentrations across pooled sampling locations was lowest in the spring (377 ± 124 CFU 100 mL^−1^), with significant (*P <* 0.001) seasonal differences observed for the summer (1,217 ± 297 CFU 100 mL^−1^) and fall (1,785 ± 428 CFU 100 mL^−1^) (Fig. 2B).

Although the forest site SN-24 had a lower volume-weighted average concentration of *Acinetobacter* species during the summer (1,056 ± 568 CFU 100 mL^−1^) and fall (740 ± 1,578 CFU 100 mL^−1^) relative to both the tributary (1,183 ± 448 and 2,367 ± 573 CFU 100 mL^−1^) and agricultural drainages (1,329 ± 588 and 1,625 ± 587 CFU 100 mL^−1^) in the same periods, all sites had similar volume-weighted concentrations (Tributary: 380 ± 297 CFU 100 mL^−1^; Drains: 376 ± 128 CFU 100 mL^−1^; Forest: 373 ± 276 CFU 100 mL^−1^) in the spring. Moreover, no significant differences across watershed groups were detected across the overall sampling period. Due to a limited number of raw drinking water intake and beach water samples, the seasonal prevalence and comparative data were not included in this analysis.

### *Acinetobacter* species detection and cell concentration in wastewater effluent

To assess the efficacy of chlorination against *Acinetobacter* species, we tested WWTP effluent samples at three stages prior to discharge of the effluent into the regional watershed. Using a genus-specific PCR assay, we detected *Acinetobacter* species in all samples collected from pre-chlorinated, chlorinated, and de-chlorinated effluent samples (Fig. 2C). The average volume-weighted cell concentration in pre-chlorinated effluent samples contained significantly (*P* < 0.05) higher concentrations (12,553 ± 5,727 CFU 100 mL^−1^) compared to chlorinated samples (44 ± 37 CFU 100 mL^−1^) and the cell concentration (584 ± 495 CFU 100 mL^−1^) in de-chlorinated samples (Fig. 2D). The overall log_10_ reduction of *Acinetobacter* species in the wastewater chlorination process was a net 1.3 reduction.

### ACB complex detection and occurrence in various water sources

**Fig 3:**
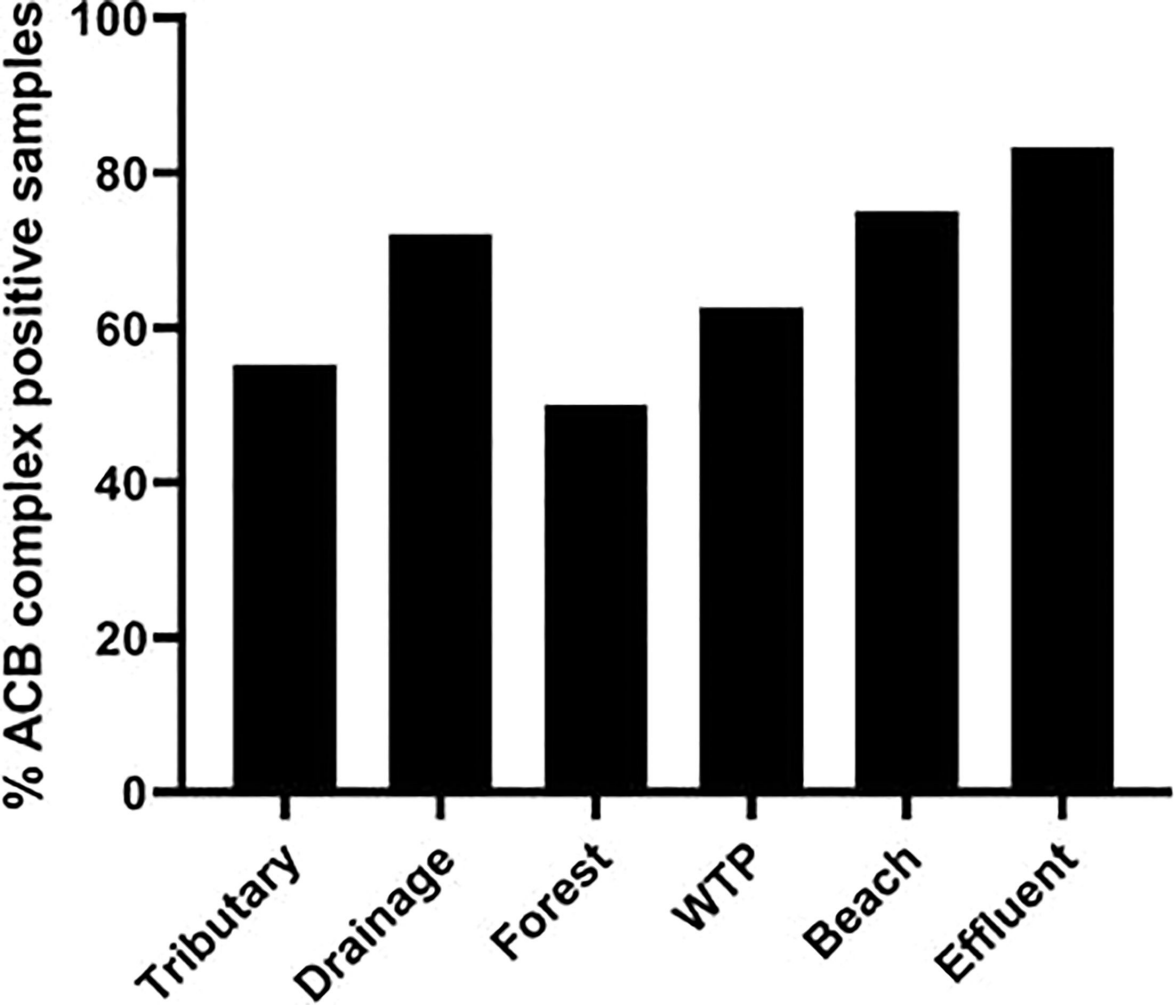
Percent of ACB complex-positive samples collected from various aquatic sources. ACB complex in agricultural (tributary and drainage), forest, WTP intake, beach and WWTP (pre-chlorinated, chlorinated, and de-chlorinated) samples.

To investigate the rate of ACB complex prevalence at all sampling locations, we further identified *Acinetobacter*-positive isolates to the species level. Overall, ACB complex were detected at all sampling locations, including the agricultural watershed, raw drinking water intake source, beach, and WWTP effluent samples at a relatively high (66%; *n* = 104) frequency. Notably, among all sampling sites, the ACB complex was detected at the highest (83%) frequency in all stages of the WWTP effluent samples, compared to agricultural samples including tributaries (57%), agricultural drains (72%), and forest/wetland (50%) (Fig. 3) sites. Similar rates of prevalence were observed in raw drinking water intake (63%) samples. Moreover, in beach water samples, a frequent (75%) detection of ACB complex was recorded, although limited samples were collected at this location and no statistical significance was observed.

### Spatial-temporal specific species-level prevalence and distribution analysis

**Fig 4:**
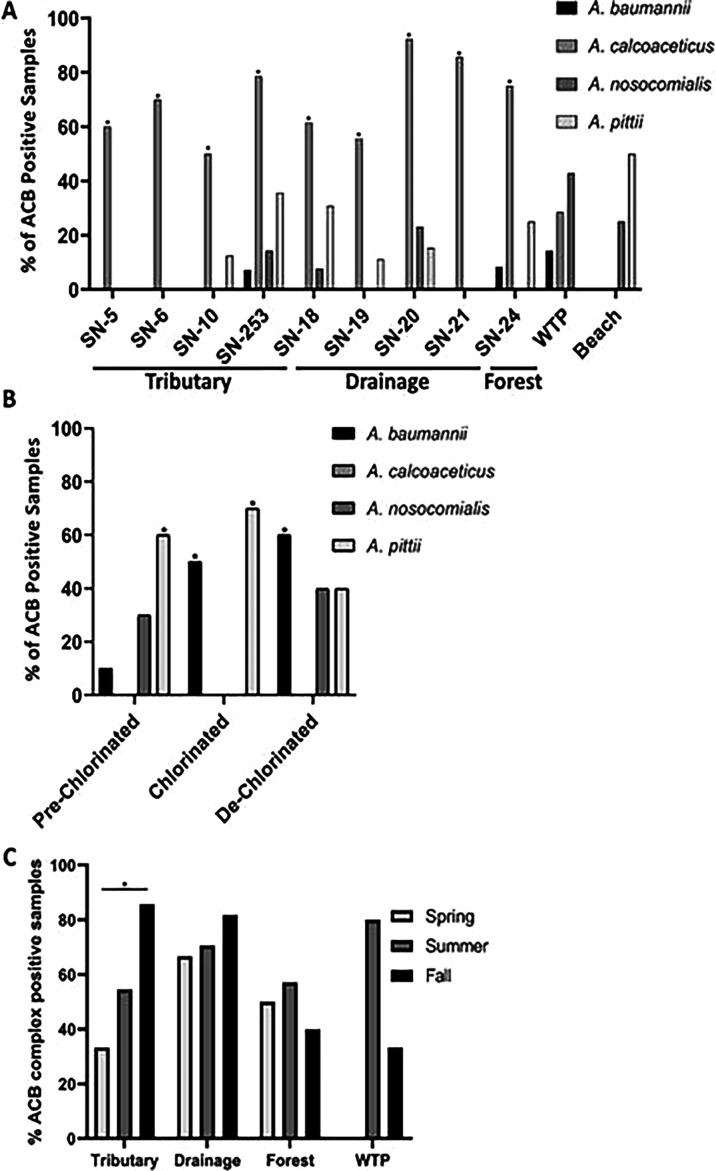
(A) percent of ACB complex detected in surface water samples collected from agricultural (tributary and drainage), forest, raw drinking intake, and beach. (B) percent ACB complex detected in WWTP samples. (C) seasonal rate of prevalence of ACB complex in tributary, agricultural drainage, forest, and WTP raw intake source samples.

Further ACB complex-specific results showed that, among agricultural and forest sites, *A. baumannii* was only detected at agricultural site SN-253 (7%, *n* = 1) and forest site SN-24 (8%, *n* = 1). *A. pittii* was detected sporadically at sites SN-253 (36%, *n* = 4), SN-18 (3%, *n* = 4), SN-24 (25%; *n* = 3), SN-20 (15%; *n* = 2), SN-10 (13%; *n* = 1), and SN-19 (11%; *n* = 1). Meanwhile, *A. nosocomialis* was only detected at sites SN-20 (23%; *n* = 3), SN-253 (14%; *n* = 2), and SN-18 (8%; 1/13). However, *A. calcoaceticus* was detected with high frequency at all sampling sites including SN-20 (92%; *n* = 12), SN-21 (86%; *n* = 6), SN-253 (79%; *n* = 11), SN-24 (75%; *n* = 9), SN-6 (70%; *n* = 7), SN-18 (62%; *n* = 8), SN-5 (60%; *n* = 6), SN-19 (56%; *n* = 5), and SN-10 (50%; *n* = 4) (Fig. 4A).

In raw drinking water intake source samples, *A. nosocomialis* (43%), *A. calcoaceticus* (29%), and *A. baumannii* (14%) were detected at a variable frequency. However, in beach samples, *A. pittii* was detected at both ankle and knee (50% each) depths, while *A. nosocomialis* was only detected in knee depth (50%) samples (Fig. 4A). Interestingly, *A. baumannii* and *A. calcoaceticus* were not detected in beach samples.

Moreover, in WWTP samples, *A. baumannii* was identified more frequently in de-chlorinated (60%) and chlorinated (50%) than pre-chlorinated (10%) as compared to *A. pittii* in chlorinated (70%) and pre-chlorinated (60%) than de-chlorinated (40%) samples. Interestingly, *A. nosocomialis* was only detected in the pre-chlorinated (30%) and de-chlorinated (40%) samples. Meanwhile, *A. calcoaceticus* was not detected in any of these samples (Fig. 4B).

Temporal prevalence and distribution of ACB complex were assessed in agricultural watershed sites. The ACB complex was detected frequently in fall samples from tributary (86%) and agriculture drainage (82%) watershed classes (Fig. 4C). Tributary sites showed a significant seasonal increase (from 33% to 86%) between spring and fall samples. Agricultural drainage samples recorded a non-significant seasonal increase (from 67% to 82%). Forest site 24 exhibited a slight increase in the ACB complex from spring to summer (from 50% to 57%) and a fall decrease (40%). Raw drinking water intake source sampling was conducted only in summer (80%) and fall (33%) and showed comparable ACB complex prevalence to other sources, with a decrease from summer to fall.

## DISCUSSION

Regional watersheds represent understudied aquatic environments for the prevalence of potentially pathogenic *Acinetobacter* species. Our previous studies have demonstrated the adaptability of *A. baumannii*, the most prominent pathogenic species of the ACB complex, to variable temperature, growth conditions, and media (31). To assess ACB complex prevalence in diverse environments, we employed various incubation conditions and selective growth media to enhance the broad recovery of ACB complex from agricultural drainages, tributaries, forest, raw drinking water intake, recreational beach water, and wastewater treatments.

*Acinetobacter*, a widely prevalent bacteria in the environment (12), was examined in various agricultural watershed sources, revealing a high prevalence in tributary, agricultural drainage, forest, and beach surface water samples. While many *Acinetobacter* species are not recognized as pathogens, the ACB complex, nowadays clinically important are only MDR/PDR *A. baumannii* presents significant risks to human health due to antimicrobial resistance and opportunistic infections (38).

The enumeration of putative *Acinetobacter* colonies on selective media revealed varying cell concentrations across sites, environments, seasons, and sampling dates. Significantly higher overall concentrations were noted in the fall, consistent with previous findings of elevated waterborne pathogen frequencies (29, 39). Seasonal differences observed could be influenced by increased summer air temperatures leading up to the fall, regional manure applications, and precipitation-induced land runoff. Although our culturing methods captured both pathogenic and non-pathogenic species, population diversity beyond the ACB complex was not explored and caused more confusion to this problem.

Among several, some *Acinetobacter* species are pathogenic and, therefore, species-level identification was performed to identify the presence of the ACB complex in water samples. The ACB complex was detected in all samples collected from agricultural (tributary, drainage), forest, raw drinking intake, and recreational beach waters. We then investigated the ACB complex species distribution across agricultural watershed sites, detecting *A. calcoaceticus* at a high prevalence in agricultural and forest sampling locations. Previous studies have identified *A. calcoaceticus* as a common environmental soil and water microbe, possibly explaining its prevalence in agriculture and forest water samples (40, 41). While *A. calcoaceticus* poses low pathogenic risk, it serves as a significant potential reservoir for antibiotic resistance genes transferable to other *Acinetobacter* species (42). *A. baumannii* was infrequently detected in both tributary waters and the forest site, and its appearance may be the result of nearby human activities such as improper waste disposal along roads near the sampling location (28). Additionally, *A. pittii* was found across all water types examined except SN-1, possibly associated with previous documentation of this pathogen in environmental water sources (43). Instead, *A. baumannii*, *A. calcoaceticus*, and *A. nosocomialis* were detected at the raw drinking water intake source, aligning with previous findings in similar environments (44, 45).

WWTPs are recognized as potential aggregators of human pathogens and antibiotic-resistant bacteria (ARB) (46), and *Acinetobacter* occurrence in WWTPs has previously been documented (18, 20). However, systematic monitoring of *Acinetobacter* species throughout the wastewater chlorination process has been sparce. This study detected *Acinetobacter*, as well as ACB complex, presence across all chlorination stages. Pre-chlorinated wastewater showed elevated *Acinetobacter* concentrations, which has been previously associated with humans and biological flocs (47). Significantly lower concentrations of *Acinetobacter* were detected in chlorinated samples. However, no significant increase was observed between the chlorinated and de-chlorinated samples though ACB complex detection in de-chlorinated effluent was consistent with previous observations (18). In the decontamination process, there was a 1.3 log_10_ reduction of *Acinetobacter* species, lower than the expected chlorine efficiency (2–6 log_10_) against bacterial pathogens (48), suggesting potential resistance to decontamination. Further investigation in the affected waterbody is needed to evaluate immediate downstream impacts of potential pathogen release in treated effluent.

To evaluate chlorination efficacy, we examined treatment stages for the presence of ACB complex species from wastewater. Although *A. calcoaceticus* is not typically found in wastewater, occasional detection in treated samples has been reported (49, 50). Conversely, *A. pittii* was consistently detected throughout effluent treatment stages, including pre-chlorinated and chlorinated samples, whereas *A. nosocomialis* was found in pre-chlorinated and de-chlorinated samples only. This persistence may be attributed to a viable non-culturable state induced by chlorine or residual chlorine hindering recovery capabilities, as observed with *A. baumannii* in similar environments (31, 51). Overall, chlorination may be less effective against ACB complex species, allowing them to persist in discharged effluent.

Unlike other members of the ACB complex, *A. baumannii* detection increased with each treatment stage, being most prevalent in de-chlorinated effluent samples. This suggests that wastewater chlorination not only fails to eliminate *A. baumannii* effectively but also may also favor its growth over other bacterial species. Consistent with previous reports, *A. baumannii* isolation from WWTP effluents using chlorine disinfection has been documented (52, 53). Additional studies suggest that disinfectant use, including chlorine, may promote the selection of antibiotic-resistant bacteria like *A. baumannii*, inducing antibiotic resistance genes in *A. baumannii* to facilitate disinfectant extrusion (22, 54). Exploring alternative disinfection technologies could be considered to better mitigate *A. baumannii* proliferation. While sodium hypochlorite is cost-effective, its efficacy is limited by organic matter and pH (55). Tracking *A. baumannii* contamination sources and dispersion is essential for optimizing environmental control measures.

In conclusion, this study sheds light on the prevalence of ACB complex and emerging *Acinetobacter* species in regional watersheds, raw drinking water intakes, and urban recreational waters in eastern Ontario, while also highlighting wastewater effluent as a potential contamination source for regional watersheds. Given reported community-acquired infections, assessing the health risks posed by ACB complex in regional watersheds is crucial. Our findings reveal consistent detection of ACB complex across all water types and seasons, with higher concentrations in the fall. Moreover, our study demonstrates the persistence of ACB complex species throughout WWTP chlorination processes, indicating WWTPs as potential secondary habitats beyond hospital settings. Disinfection techniques may not effectively remove ACB complex species, warranting further antibiotic resistance assays to understand selection mechanisms. Therefore, alternative effluent disinfection methods could be explored to mitigate ACB complex propagation into downstream water sources and reduce community-acquired *Acinetobacter* infection risks. The occurrence of ACB complex in aquatic niches underscores the importance of natural water and wastewater effluent monitoring.

## SUPPLEMENTAL MATERIAL

Fig. S1 spectrum.01509-24-s0001.tif Colony morphology of *Acinetobacter* species on CHROMagar and LAM at 37°C and 30°C.

ASM does not own the copyrights to Supplemental Material that may be linked to, or accessed through, an article. The authors have granted ASM a non-exclusive, world-wide license to publish the Supplemental Material files. Please contact the corresponding author directly for reuse.
