# Supplementary figures and images for: Acinetobacter calcoaceticus-baumannii complex prevalence, spatial-temporal distribution, and contamination sources in Canadian aquatic environments
Source: Microbiol Spectr. 2024 Sep 6;12(10):e01509-24. doi: 10.1128/spectrum.01509-24 (PMC11449026; doi:10.1128/spectrum.01509-24)

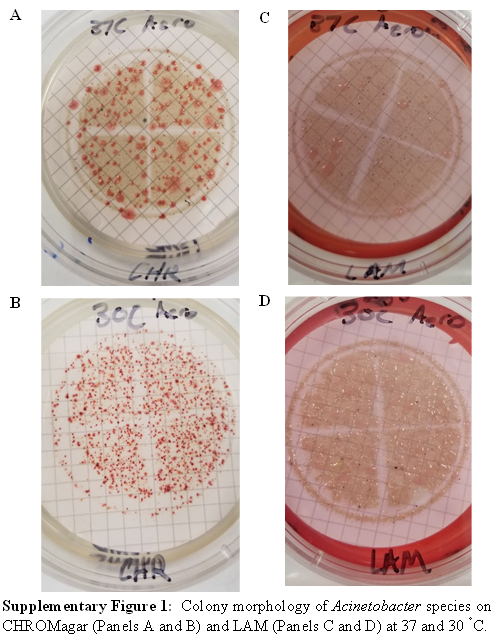

Supplement: Fig. S1 — Colony morphology of Acinetobacter species on CHROMagar and LAM at 37°C and 30°C. [file spectrum.01509-24-s0001.tif]
